# Optimizing Nodal, Wnt and BMP signaling pathways for robust and efficient differentiation of human induced pluripotent stem cells to intermediate mesoderm cells

**DOI:** 10.3389/fcell.2024.1395723

**Published:** 2024-06-03

**Authors:** Esmeralda Magro-Lopez, Elena Vazquez-Alejo, María de la Sierra Espinar-Buitrago, María Ángeles Muñoz-Fernández

**Affiliations:** ^1^ Molecular Immuno-Biology Laboratory, Immunology Section, Hospital General Universitario Gregorio Marañón (HGUGM), Madrid, Spain; ^2^ Instituto de Investigación Sanitaria Gregorio Marañón (IiSGM), Madrid, Spain; ^3^ Biomedical Research Networking Center in Bioengineering, Biomaterials and Nanomedicine (CIBER-BBN), Instituto de Salud Carlos III (ISCIII), Madrid, Spain

**Keywords:** hiPSC, mesoderm progenitors, intermediate mesoderm, Nodal, Wnt, BMPs, 3D organoids, urogenital organoids

## Abstract

Several differentiation protocols have enabled the generation of intermediate mesoderm (IM)-derived cells from human pluripotent stem cells (hPSC). However, the substantial variability between existing protocols for generating IM cells compromises their efficiency, reproducibility, and overall success, potentially hindering the utility of urogenital system organoids. Here, we examined the role of high levels of Nodal signaling and BMP activity, as well as WNT signaling in the specification of IM cells derived from a UCSD167i-99-1 human induced pluripotent stem cells (hiPSC) line. We demonstrate that precise modulation of WNT and BMP signaling significantly enhances IM differentiation efficiency. Treatment of hPSC with 3 μM CHIR99021 induced TBXT+/MIXL1+ mesoderm progenitor (MP) cells after 48 h of differentiation. Further treatment with a combination of 3 μM CHIR99021 and 4 ng/mL BMP4 resulted in the generation of OSR1+/GATA3+/PAX2+ IM cells within a subsequent 48 h period. Molecular characterization of differentiated cells was confirmed through immunofluorescence staining and RT*-*qPCR. Hence, this study establishes a consistent and reproducible protocol for differentiating hiPSC into IM cells that faithfully recapitulates the molecular signatures of IM development. This protocol holds promise for improving the success of protocols designed to generate urogenital system organoids *in vitro*, with potential applications in regenerative medicine, drug discovery, and disease modeling.

## Introduction

The intermediate mesoderm (IM) is a band section of mesodermal tissue lying between the paraxial mesoderm (PSM) and the lateral plate mesoderm (LPM) (Nájera and Weijer, 2023). It serves as the precursor for the urogenital system, including the kidneys, gonads, and their associated ducts (Saxón and Sariola, 1987). During gastrulation, the IM originates from mesoderm progenitors (MP) formed within the posterior primitive streak (PPS) (Saxón and Sariola, 1987; Nájera and Weijer, 2023). Gastrulation is a crucial stage in embryonic development, where the single-layered blastocyst transforms into a tri-layered structure, the ectoderm, mesoderm, and endoderm. The primitive streak, a transient structure formed during gastrulation, plays a key role in this process. Cells from the epiblast (outer layer) undergo epithelial-to-mesenchymal transition and migrate inwards through the primitive streak. The initial wave displaces the hypoblast cells to form the definitive endoderm (DE) (future gut lining). The second wave populates the space between the epiblast and endoderm, giving rise to the mesoderm layer. This mesoderm further differentiates into five subpopulations: paraxial, intermediate, lateral plate, cardiogenic, and notochordal mesoderm. Once the mesoderm is formed, the remaining epiblast cells stop migrating and become the ectoderm (outer layer) (Mouse development, 2007; Ghimire et al., 2021; Schoenwolf et al., 2021; Zhai et al., 2022).

Intricate signaling pathways, like Nodal, WNT, and BMP, activate specific sets of genes that dictate the fate of each cell, ultimately shaping the developing organism (Perrimon et al., 2012). The Nodal pathway plays a central role in specifying mesoderm fate (Schier, 2003; Ben-Haim et al., 2006). Traditionally, Nodal signaling has been viewed as a graded morphogen, where high levels trigger the formation of the endoderm layer and lower levels promote mesoderm development (Schier, 2003; Zorn and Wells, 2009). WNT signaling is crucial in mesoderm development. Studies have demonstrated its necessity for both initiating mesoderm formation and maintaining the pool of posterior mesodermal progenitors (Lindsley et al., 2006; Martin and Kimelman, 2008; Tanaka et al., 2011; Blauwkamp et al., 2012; Przybyla et al., 2016). BMP signaling also plays a critical role, with lower levels promoting the formation of IM, while higher levels favor the development of the LPM (Tonegawa and Takahashi, 1998; James and Schultheiss, 2005; Knarston et al., 2020).

Technical and ethical limitations pose a significant challenge to directly studying early human development. However, human pluripotent stem cells (hPSC) offer powerful tool to overcome these limitations. Recent studies have demonstrated the generation of various cell types from hPSC, including IM-derived cells from hPSC (Yucer et al., 2021; 2017; Miyazaki et al., 2018; Knarston et al., 2020; Bejoy et al., 2022; Gong et al., 2022). This ability of hPSC to recapitulate different stages of human embryonic development, mimicking the natural developmental sequence, has revolutionized our understanding of human biology and disease. This has led to significant advancements in disease modeling, drug discovery, and regenerative medicine research (Thomson et al., 1998; Takahashi et al., 2007; Zhu and Huangfu, 2013; Kim et al., 2020). hPSC hold another valuable application in developing organoids for modeling genetic disorders. This ability to create patient-specific organoids reflects the growing recognition of genetic diversity and inter-individual variability in human biology and disease (Perez-Lanzon et al., 2018). The UCSD167i-99-1 hPSC line used in this study serves as a well-defined system for studying these factors. Its extensive genetic characterization makes it a reliable tool for investigating the impact of genetics on cellular differentiation (Panopoulos et al., 2017).

Urogenital organoids derived from hPSC are emerging as powerful models for studying human-specific aspects of urogenital development, disease and drug discovery. Thus, understanding the molecular mechanisms underlying IM induction *in vitro*, is crucial for elucidating organogenesis and tissue patterning during embryonic development. Several methods have been developed to derive mature tissues from the IM lineage using hPSC. These protocols have successfully generated tissue, such as fallopian tube epithelium (Yucer et al., 2021; 2017), endometrial stromal fibroblasts (Miyazaki et al., 2018), podocytes (Bejoy et al., 2022), Müllerian duct-like cells (Gong et al., 2022) or gonad and testis cell (Knarston et al., 2020; Yucer et al., 2017 showed the generation of TBXT+ (T-box transcription factor T, protein) (BRACHYURY)/MIXL1+ (Mix paired-like homeobox, protein) PPS and OSR1+ (Odd-skipped related transcription factor 1, protein)/GATA3+ (GATA binding protein 3, protein)/PAX2+ (paired box 2, protein) IM cells. In their study, human induced pluripotent stem cells (hiPSC) were initially exposed to 100 ng/mL human recombinant Activin A and 3 μM CHIR99021 for 48 h to differentiate into mesoderm, followed by IM induction using 100 ng/mL BMP4 and 3 μM CHIR99021 for 48 h. Knarston et al., 2020 described PPS differentiation with 3 μM CHIR99021 for 96 h and IM lineage OSR1+/LHX1+ (LIM homeobox 1, protein)/PAX2+ with 10 ng/mL BMP4, 1 μg/mL Heparin and 200 ng/mL FGF9 for 72 h. Bejoy et al., 2022 have reported an accelerated method by differentiating hPSC into MIXL1+ primitive streak cells with 100 ng/mL human Activin A and 3 μM CHIR99021 for 48 h, and IM PAX8+(paired box 8, protein) cells with 8 μM CHIR99021 for 72 h. Gong et al., 2022 treated hPSC with 5 μM CHIR99021 for 36 h, identifying successful induction of TBXT+/MIXL1+ mesendodermal cells; then 100 ng/mL basic fibroblast growth factor (bFGF) and 10 nM Retinoic acid (RA) for OSR1+/PAX2+/LHX1+IM cell differentiation for 72 h.

While these studies have demonstrated the successful generation of hPSC-derived IM cells, the differentiation strategies employed exhibit substantial variability between protocols, with variations in the choice of morphogens and the concentrations of the signaling molecules. Meanwhile, to accurately recapitulate IM development, hPSC differentiation should emulate the natural sequence of molecular signals that occur during embryogenesis as closely as possible.

In this sense, refining and optimizing the existing differentiation methodology to enhance the efficiency of IM cell generation would significantly increase the reproducibility and success of protocols to form urogenital system organoids *in vitro*.

For this purpose, we adopted the strategy outlined by Yucer et al., 2017 and investigated the roles of high levels of Nodal and BMP signaling activity, along with WNT signaling, in the determination of IM cells derived from a UCSD167i-99-1 hiPSC line, obtained from the WiCell Research Institute.

Here, we report a significant yet simple modification of the conventional methods, enabling IM development to faithfully recapitulate the molecular features described in embryological literature. We demonstrate that IM cells can be generated from a UCSD167i-99-1 hPSC line, addressing these features by suppressing the Nodal signaling during the mesoderm step and employing low concentrations of BMP4 for IM generation. Finally, our approach enhances the efficacy of IM differentiation and establishes a robust and reproducible protocol for differentiating hiPSC into IM cells.

## Materials and methods

### Maintenance of hiPSC culture

hiPSC line UCSD167i-99-1, which has been previously characterized (Panopoulos et al., 2017), was obtained from the WiCell Research Institute (Madison, WI). UCSD167i-99-1 cells were cultured following the manufacturer’s guidelines between passages 19 and 40. hiPSC were maintained as colonies in feeder-free conditions on hPSC-qualified Matrigel™ (cat.#354277, Corning) in mTeSR™1 (cat.#85850, Stem Cell Technologies) or mTeSR™ Plus medium (cat.#100-0276, Stem Cell Technologies) in 6-well Nunclon Delta surface plates (cat.#140675, Thermofisher Scientific). Cultures were maintained in an undifferentiated state in a 5% CO_2_/air environment. Medium was replaced daily, and cells were passaged every 4–6 days using Gentle Cell Dissociation Reagent (cat.#7174, Stem Cell Technologies) at a 1:6 split ratio. hiPSC were maintained for at least three passages before differentiation.

### Induction of MP cells

hiPSC were directed into MP cells following a modified version of the protocol described by Yucer et al. (Yucer et al., 2017). When hiPSC reached approximately 70% confluence, medium was replaced with DMEM/F12 (cat.#11320-033, Gibco, Thermofisher Scientific) supplemented with 1% Glutamax (cat.#13462629, Gibco, Thermofisher Scientific), 1% penicillin and streptomycin (cat.#15070063, Thermofisher Scientific), 2% Fetal Bovine Serum (FBS) (cat.#S0615, Sigma-Aldrich) and 10 μM Y27632 (a ROCK inhibitor) (cat.#72304, Stem Cell Technologies). Cells were then treated with 100 ng/mL human recombinant activin A (cat.#338-AC-010/CF, R&D Systems) or 3 μM CHIR99021 (cat.#4423, R&D Systems) for 24, 48 and 72 h, accordingly.

### Induction of IM cells

To induce IM cells, we refined the method described by Yucer et al. (Yucer et al., 2017). MP cells were washed once in 1X PBS, dissociated into single cells with trypsin, and the reaction was halted with stop medium (IMDM medium (cat.#I659, Sigma-Aldrich) supplemented with 50% fetal bovine serum, 2 mM Glutamax, 1% penicillin-streptomycin and 30 ng/mL DNase I (cat.#260913; Sigma-Aldrich). Cells were then centrifuged for 5 min at 300 *g* and washed twice carefully in an excess of DMEM/F12 medium. Cells were then plated at a 1:6 split ratio (wells from 6-well dish: wells in 24-well dish) on plastic coverslips (cat.#11846933-174969, Thermofisher Scientific) onto 24-well tissue culture plates (cat.#353047, Corning), as previously described by McCracken et al. (McCracken et al., 2011). Cultures were incubated in DMEM/F12 supplemented with 1% Glutamax, 1% penicillin and streptomycin, 0.1 mM non-essential amino acids (cat.#11140050, Thermofisher Scientific), 0.55 mM 2-mercaptoethanol (cat.#21985–023, Thermofisher Scientific), 10% knockout serum replacement (cat.#10828–010, Stem Cell Technologies) and 10 μM Y27632. Cells were treated with 3 μM CHIR99021 and 100 ng/mL BMP4 (cat.#314-BP-010, R&D Systems) for 48 h, accordingly.

### Indirect immunofluorescence

#### Coating coverslips for hiPSC and embryonic tissue immunostaining

Sterilized glass coverslips (cat.# 11856933, Thermofisher Scientific) were placed into wells of 24-well plates and treated with fibronectin (0.33% vol/vol) (cat.#1918-FN, R&D Systems) for 2 h at RT. Subsequently, coverslips were washed twice in 1X PBS and allowed to dry in the laminar flow hood. Then, coverslips were coated with Matrigel™ overnight. hiPSC were plated at a 1:24 split ratio (wells from 6-well dish: wells in 24-well dish) onto Matrigel™-coated glass coverslips and maintained in mTeSR™1 medium for 24 h before hiPSC immunostaining. For MP immunostaining, the medium was replaced with induction medium and cells were incubated for an additional 48 h before staining. For IM immunostaining, 70,000-140,000 cells per well were plated onto fibronectin-coated 24-well plates and maintained in induction medium for 48 h before staining.

### Immunostaining

Coverslips with cells were rinsed in 1X PBS and fixed in 4% PFA (cat.#1004968350, Sigma-Aldrich) for 10 min at RT. Fixed cells were washed three times in 1X PBS and permeabilized with 0.1% Triton X-100 (cat.#T8787, Sigma-Aldrich) diluted in blocking buffer for 5 min at RT. Permeabilized cells were washed three times in 1X PBS and then blocked with 3% goat serum (cat.#G9023, Sigma-Aldrich) diluted in 1X PBS for 30 min at RT. Cells were incubated with primary antibodies diluted in blocking solution overnight at 4°C. After washing three times in 1X PBS, cells were incubated in secondary antibodies diluted in blocking solution for 2 h at RT. Cells were washed three times in 1X PBS and nuclei were counterstained with DAPI. Coverslips were mounted in slides with ProLong Diamond (cat.#P36961, Thermofisher Scientific). Cell images were acquired using a Leica TSC SPE confocal microscope (Leica Microsystems). Fluorescence was quantified by counting from >200 cells for each experimental condition using Adobe photoshop. Fluorescence was analyzed using LASX software (Leica Microsystems).

Immunostaining was performed using the following primary antibodies: mouse anti- POU5F1 (1:200; sc-5279, Santa Cruz), mouse anti- NANOG (1:200; sc-293121, Santa Cruz), rabbit anti- SOX2 (1:100; A5-16399, Thermofisher), rabbit anti-TBXT (1:500; ab20680, Abcam), rabbit anti- MIXL1 (1:200; BS-12350R, Thermofisher Scientific), rabbit anti- PAX2 (1:500; ab150391, Abcam) and mouse anti- OSR1 (1:100; sc-376545, Santa Cruz); and the following secondary antibodies: goat anti-Mouse Alexa Fluor 488 (1:300; A-11029, Thermofisher Scientific) and goat anti-rabbit-Alexa Fluor 546 (1:500; A-11035, Thermofisher Scientific).

### Quantitative real-time RT-PCR (RT-qPCR) analysis

Total RNA was isolated using the Qiagen RNeasy Mini kit (cat.#74014; Qiagen) following the manufacturer’s instructions. The concentration of total RNA was measured using a NanoDrop (Thermofisher Scientific). cDNA was synthesized using the High-Capacity cDNA kit (cat.#4368814; Thermofisher Scientific). Real-time qPCR was performed using the PowerUpSYBR Green mix (cat.#A25776, Thermofisher Scientific) on the Stratagene*™* MX3005P (Thermofisher Scientific). Absolute quantification was performed using a standard curve of serial diluted genomic DNA and normalized to β-Actin. Relative quantification was performed by comparing the samples to undifferentiated hiPSC as a reference control, and was determined using the ΔΔCt method.Primer sequences used in this study were as follows:

*β-ACTIN* (Actin beta, cytoskeletal protein) [Forward: 5′- TGG​CAC​CAC​ACC​TTC​TAC​AAT​GA, Reverse: 5′- CAG​CCT​GGA​TAG​CAA​CGT​ACA​T]; *POU5F1* (POU class 5 homeobox 1, marker of pluripotency) [Forward: 5′-ACC​CAC​ACT​GCA​GCA​GAT​CA, Reverse: 5′- CCA​CAC​TCG​GAC​CAC​ATC​C]; *NANOG* (Nanog homeobox, marker of pluripotency) [Forward: 5′ACA​ACT​GGC​CGA​AGA​ATA​GCA, Reverse: 5′- GGT​TCC​CAG​TCG​GGT​TCA​C]; *SOX2* (SRY-box transcription factor 2, marker of pluripotency) [Forward: 5′GGG​GGA​ATG​GAC​CTT​GTA​TAG, Reverse: 5′- GCA​AAG​CTC​CTA​CCG​TAC​CA]; *TBXT* (T-box transcription factor T) (*BRACHYURY)*, marker of mesoderm) [Forward: 5′GCT​GTG​ACA​GGT​ACC​CAA​CC, Reverse: 5′- CAT​GCA​GGT​GAG​TTG​TCA​GAA]; *MIXL1* (Mix paired-like homeobox, marker of mesoderm) [Forward: 5′GGT​ACC​CCG​ACA​TCC​ACT​T, Reverse: 5′- GCC​TGT​TCT​GGA​ACC​ATA​CCT]; *SOX17* (SRY-box transcription factor 17, marker of definitive endoderm) [Forward: 5′ACG​CCG​AGT​TGA​GCA​AGA, Reverse: 5′- TCT​GCC​TCC​TCC​ACG​AAG]; *FOXA1* (forkhead box A1, marker of definitive endoderm) [Forward: 5′GCA​ATA​CTC​GCC​TTA​CGG​CT, Reverse: 5′- TAC​ACA​CCT​TGG​TAG​TAC​GCC]; *OSR1* (odd-skipped related transcription factor 1, marker of intermediate mesoderm) [Forward: 5′GGA​CCT​CTG​CGG​AAC​AAG, Reverse: 5′- TGC​AGG​GAA​GGG​TGG​ATA]; *GATA3* (GATA binding protein 3, marker of intermediate mesoderm) [Forward: 5′CTC​ATT​AAG​CCC​AAG​CGA​AG, Reverse: 5′- GTC​TGA​CAG​TTC​GCA​CAG​GA]; *PAX2* (paired box 2, marker of intermediate mesoderm) [Forward: 5′GAA​GTG​CCC​CCT​TGT​GTG, Reverse: 5′- TCG​TTG​TAG​GCC​GTG​TAC​TG].

### Statistical analysis

The data were represented as the mean ± standard error of the mean (SEM) of *n* = 3 biological replicates from at least three independent experiments. For statistical comparisons, unpaired Student's *t*-tests were performed to calculate *p*-values between experimental conditions and controls and a *p*-value <0.05 was considered statistically significant. Significance of the analysis of the Student’s t-test is indicated in the figures as *, *p* < 0.05; **, *p* < 0.01; ***, *p* < 0.001 and ****, *p* < 0.0001. Statistics were calculated with the Prism nine software (GraphPad Software).

## Results

**FIGURE 1:**
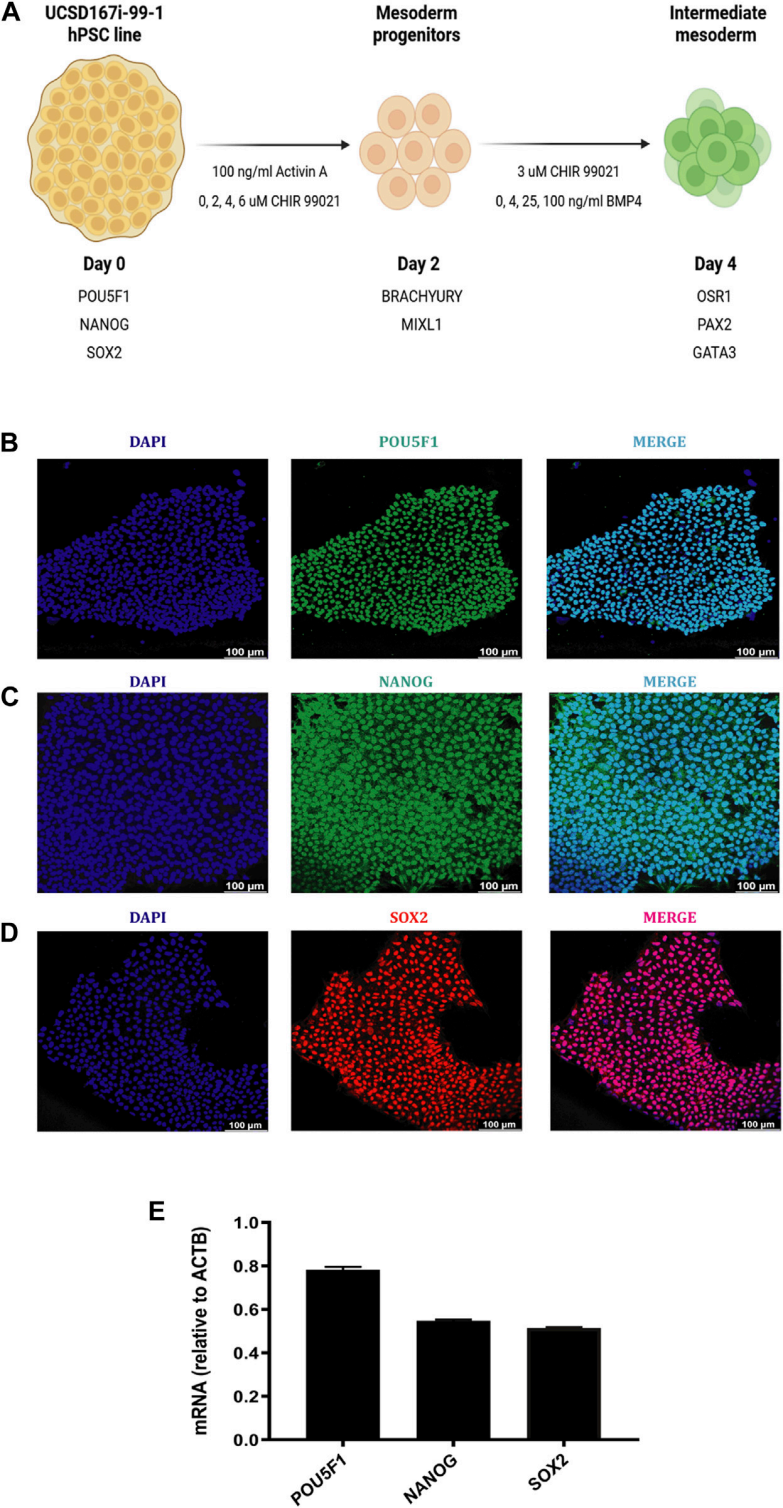
Characterization of the UCSD167i-99-1 hiPSC line. **(A)** Schematic diagram of the differentiation strategy adopted by Yucer et al., 2017 with modifications, to induce hiPSC into MP and IM cells. **(B–D)** Immunofluorescence images of hiPSC colonies stained for POU5F1 (green), NANOG (green), SOX2 (red), merged with nucleus marker DAPI (cyan). Scale bars: 100 μm. **(E)** Absolute mRNA expression levels of pluripotency markers determined by RT*-*qPCR analysis in undifferentiated hiPSC colonies. Error bars represent mean *±* SEM, *n* = 3 independent experiments, n.s. not significant, *p < *0.05*, **p < 0.01, ***p < 0.001, ****p < 0.0001. hiPSC human induced pluripotent stem cells, MP mesoderm progenitor, IM intermediate mesoderm, DE definitive endoderm.

We aimed to establish an optimized protocol for generating UCSD167i-99-1 hiPSC line-derived induced IM cells. To achieve this, we modified the differentiation protocol described by Yucer et al., 2017 (Figures 4A, B). We first optimized the conditions by differentiating hiPSC into MP cells and then into IM cells, following a modified version of the Yucer et al., 2017 strategy (Figure 1A).

The hiPSC line used in our study was UCSD167i-99-1 obtained from the WiCell Research Institute (Madison, WI). UCSD167i-99-1 line was established from skin fibroblasts of a healthy female individual. Figures 1B–D show the characteristic morphology of UCSD167i-99-1 colonies grown on feeder free culture using Matrigel and mTeSR™1 medium. UCSD167i-99-1 maintained in an undifferentiated state shows a high and homogenous expression of POU5F1+ (POU class 5 homeobox 1, protein), SOX2+ (SRY-box transcription factor 2, protein) and NANOG+ (Nanog homeobox, protein), common markers of pluripotency (Figures 1B–D). Molecular characterization by RT-qPCR showed the expression of pluripotency markers *POU5F1*, *NANOG* and *SOX2* (Figure 1E).

It has been reported that activation of WNT signaling promotes efficient MP differentiation (Lindsley et al., 2006; Tanaka et al., 2011; Blauwkamp et al., 2012; Przybyla et al., 2016). Therefore, our first step was to expose hiPSC to various concentrations of a glycogen synthase kinase 3b (GSK3β) inhibitor, CHIR99021, alone, as an agonist and potent WNT pathway activator (Blauwkamp et al., 2012; Naujok et al., 2014). To determinate the efficacy of CHIR99021 to induce the mesoderm state in the UCSD167i-99-1 hiPSC line, we tested treatments with 0, 2, 4 and 6 μM CHIR99021 for 24, 48 and 72 h and monitored the expression of MP markers, TBXT or MIXL1 (Izumi et al., 2007; Martin and Kimelman, 2008; Pereira et al., 2011; Turner et al., 2014; Faial et al., 2015; Yucer et al., 2021; 2017; Carpenedo et al., 2019), by immunofluorescence and RT-qPCR.

**FIGURE 2:**
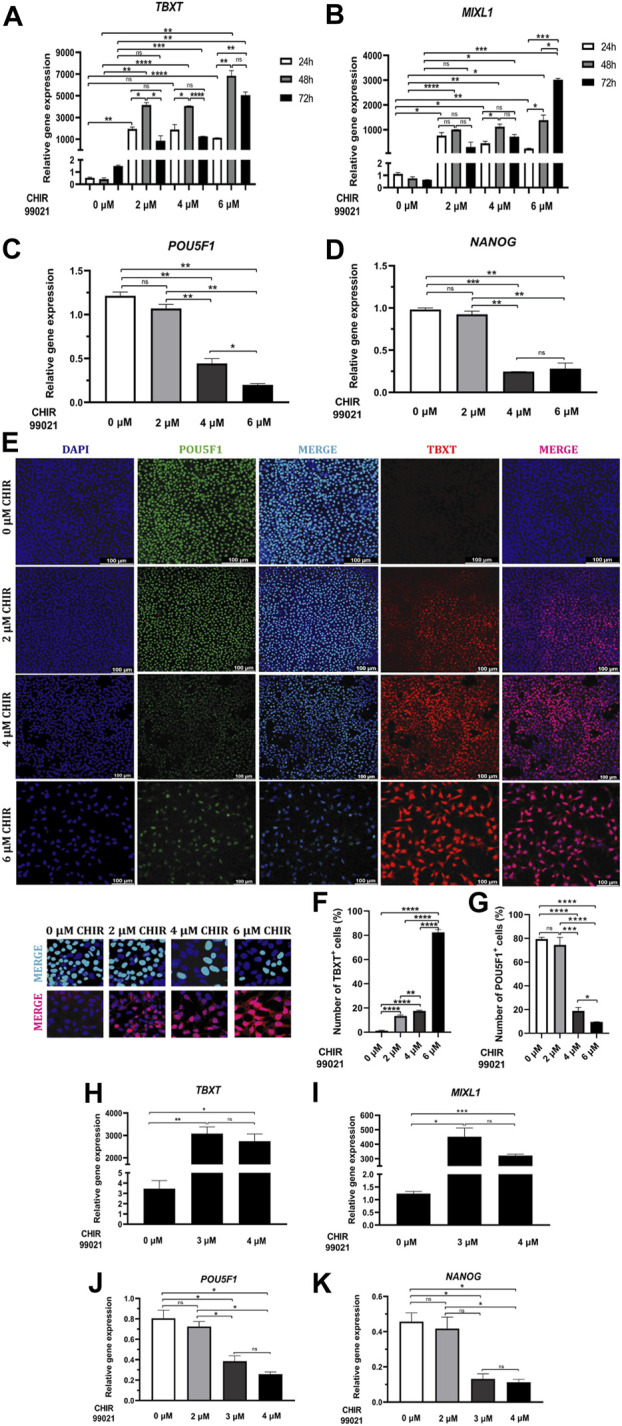
WNT-mediated MP cells differentiation. **(A, B)** Relative mRNA expression levels of MP markers *TBXT* and *MIXL1* determined by RT*-*qPCR in differentiated cells at 24, 48 and 72 h of treatment with 0, 2, 4 and 6 µM CHIR99021. **(C, D)** Relative mRNA expression levels of pluripotent markers *POU5F1* and *NANOG* determined by RT*-*qPCR in differentiated cells at 48 h of treatment with 0, 2, 4 and 6 µM CHIR99021. **(E)** Immunofluorescence images of differentiated cells stained for pluripotent marker POU5F1 (green), MP marker TBXT (red), merged with nucleus marker DAPI (cyan or magenta) at 48 h of treatment with 0, 2, 4 and 6 µM CHIR99021. Scale bars: 100 μm. High-resolution insets (×40 magnification) are presented directly below the main figure. These insets zoom in on representative cells, highlighting the details of stained merged nuclei (POU5F1 or TBXT with DAPI). This enhanced view complements the main figure, which displays images at a lower magnification (×20). **(F, G)** Quantification of TBXT and POU5F1+ cells in MP cells differentiated from hiPSC after 48 h of treatment with 0, 2, 4 and 6 µM CHIR99021. **(H, I)** Relative mRNA expression levels of MP markers *TBXT* and *MIXL1* determined by RT*-*qPCR in differentiated cells at 48 h of treatment with 0, 3 and 4 µM CHIR99021. **(J, K)** Relative mRNA expression levels of pluripotent markers *POU5F1* and *NANOG* determined by RT*-*qPCR in differentiated cells at 48 h of treatment with 0, 2, 3 and 4 µM CHIR99021. Error bars represent mean *±* SEM, *n* = 3 independent experiments, n. s not significant, *p < *0.05*, **p < 0.01, ***p < 0.001, ****p < 0.0001. Gene expression levels were determined by relative quantification compared to undifferentiated hiPSC. hiPSC human induced pluripotent stem cells, MP mesoderm progenitor, IM intermediate mesoderm, DE definitive endoderm.

Gene expression analysis revealed a significant upregulation of *TBXT* within 24 h of treatment with 2 and 6 μM CHIR99021 compared to 0 μM CHIR99021, peaked at 48 h with 2, 4, and 6 μM CHIR99021 treatments compared to control and then decreased by 72 h with 4 and 6 μM CHIR99021 treatments compared to control. Treatments with 2 and 4 μM CHIR99021 induced significantly higher levels of *TBXT* at 48 h compared to 24 and 72 h (Figure 2A).

Increased expression levels of *MIXL1* were observed significantly within 24 h of treatment with 2, 4 and 6 μM CHIR99021, compared to 0 μM CHIR99021, peaked at 48 h with all three concentrations of CHIR99021, and decreased by 72 h with 4 and 6 μM CHIR99021, compared to control. Treatment with 6 μM CHIR99021 induced significantly higher levels of *MIXL1* at 48 and 72 h, compared to 24 h (Figure 2B).

However, qPCR analysis revealed a significant downregulation of pluripotency-associated genes *POU5F1* and *NANOG* in cells treated with 4 and 6 μM CHIR99021 compared to 2 μM CHIR99021 and the untreated control group (0 μM) (Figures 2C, D).

Immunofluorescence analysis after 48 h of exposure showed that treatment >2 μM CHIR99021 induced the MP marker TBXT (Figure 2E). However, higher doses >4 μM CHIR99021 resulted in a concentration-dependent decrease in cell number (Figure 2E). Moreover, the percentage of TBXT was significantly higher with increasing concentrations of CHIR99021 (Figure 2F). To validate these findings and determine the optimal CHIR99021 concentration, we evaluated the expression levels of the MP markers *TBXT* and *MIXL1* by RT-qPCR in hiPSC exposed to 0, 3, and 4 μM CHIR99021 for 48 h (Figures 2H, I). The results revealed a significant increase in both *TBXT* and *MIXL1* expression in cells treated with three and 4 μM CHIR99021 compared to 0 μM CHIR99021. There were no statistically significant differences in the expression levels of either marker between the 3 μM and 4 μM CHIR99021 concentrations (Figures 2H, I). Similarly, while genes associated with pluripotency (*POU5F1* and *NANOG*) remained downregulated compared to the 0 μM and 2 μM treatment groups, no significant differences were found between the 3 μM and 4 μM CHIR99021 treatments (Figures 2J, K). Thus, we chose an optimal CHIR99021 concentration of 3 μM and a treatment duration of 48 h for subsequent experiments.

We also detected co-expression of the pluripotency marker POU5F1 with MP marker TBXT. However, the increase in TBXT expression is consistent with a reduction in pluripotency, as evidenced by the decreased POU5F1 staining in confocal images (Figure 2E). Figure 2G further demonstrates a statistically significant decrease in the percentage of POU5F1+ cells within the MP cell population upon treatment with increasing concentrations of CHIR99021.

**FIGURE 3:**
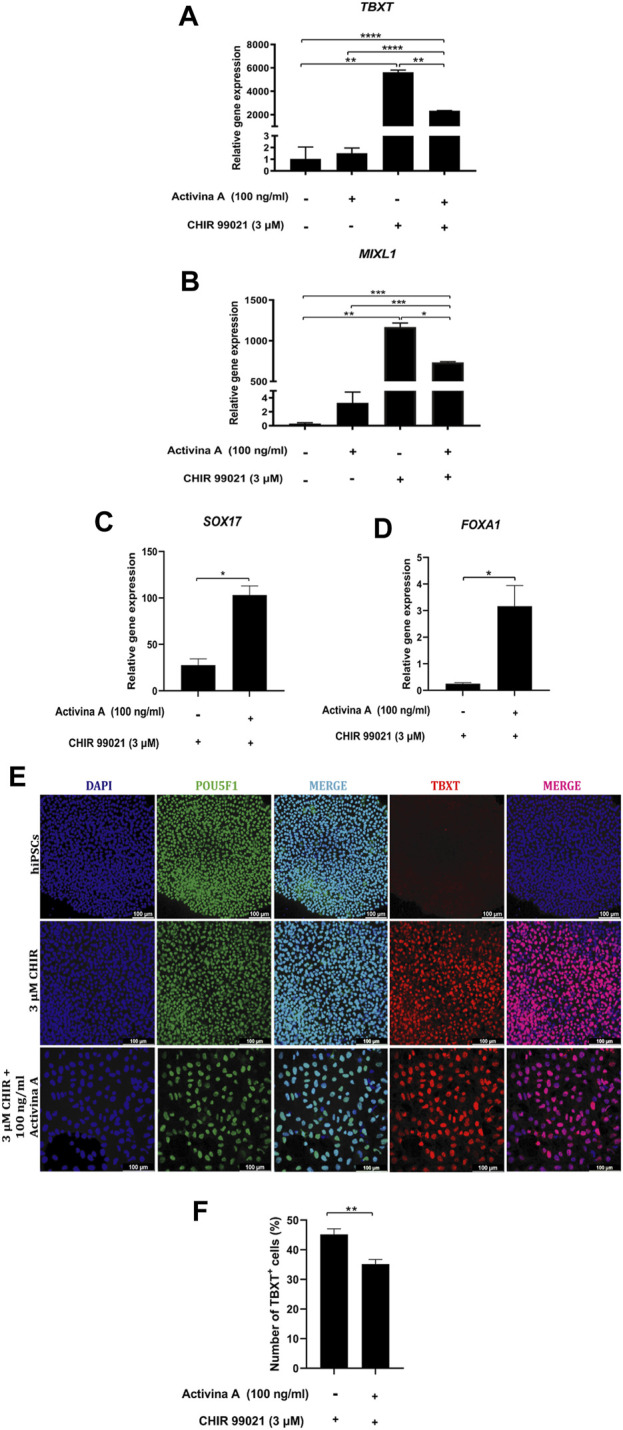
Nodal and WNT-mediated MP cells differentiation. **(A–D)** Relative mRNA expression levels of MP markers *TBXT*, *MIXL1* and DE markers *SOX17* and *FOXA1* determined by RT*-*qPCR in differentiated cells at 48 h of treatment with 3 µM CHIR99021 alone and with 100 ng/mL Activin A. **(E)** Immunofluorescence images of hiPSC colonies and differentiated cells stained for POU5F1 (green), MP marker TBXT (red), merged with nucleus marker DAPI (cyan or magenta) at 48 h of treatment with 3 µM CHIR99021 alone and with 100 ng/mL Activin A. Scale bars: 100 μm. **(F)** Quantification of TBXT + cells in MP cells differentiated from hPSC after 48 h of treatment with 3 µM CHIR99021 alone and with 100 ng/mL Activin A. Error bars represent mean *±* SEM, *n* = 3 independent experiments, n. s not significant, *p < *0.05*, **p < 0.01, ***p < 0.001, ****p < 0.0001. Gene expression levels were determined by relative quantification compared to undifferentiated hiPSC. hiPSC human induced pluripotent stem cells, MP mesoderm progenitor, IM intermediate mesoderm, DE definitive endoderm.

To assess the role of highly activated Nodal signaling in the generation of MP cells from hiPSC, we evaluated the effects of 100 ng/mL Activin A, a known agonist of Nodal signaling (Baharvand and Aghdami, 2012), alone or in combination with 3 μM CHIR99021 for 48 h. We found that the addition of Activin A to CHIR99021 significantly decreased the expression levels of *TBXT* (Figure 3A) and *MIXL1* (Figure 3B), compared to CHIR99021 alone, although resulted in increased expression levels of *SOX17* and *FOXA1*, DE markers (Séguin et al., 2008; Wang et al., 2011; Reizel et al., 2021), compared to CHIR99021 alone (Figures 3C, D).

Immunostaining analysis confirmed that undifferentiated hiPSC expressed POU5F1 but not TBXT. Treatments with CHIR99021 alone or in combination with Activin A clearly induced the expression of TBXT (Figure 3E). However, the percentage of TBXT + cells was significantly lower in Activin A in combination with CHIR99021 treatment than in CHIR99021 alone (Figure 3F). The pluripotency marker, POU5F1, was still expressed during the induction of MP cells from hiPSC (Figure 3E).

In order to evaluate the effect of BMP signaling activity on IM specification, we used different concentrations of BMP4, a key ligand in IM differentiation (James and Schultheiss, 2005), in combination with 3 μM CHIR99021, also involved in the differentiation of IM (Sepponen et al., 2017). After 48 h of exposure, CHIR99021-induced MP cells were exposed to 0, 4, 25 and 100 ng/mL BMP4 and 3 μM CHIR99021 for 48 h. We optimized the conditions for the generation of IM spheroids as described in (McCracken et al., 2011). Differentiation outcomes were assessed by immunofluorescence and qPCR analysis of known IM markers OSR1, GATA3 and PAX2 (Yucer et al., 2021; 2017).

We first verified the absence or low gene expression levels of the IM markers *OSR1*, *PAX2* and *GATA3* in MP cells by RT-qPCR (Supplementary Figures 1A–C). We also confirmed low expression of OSR1 and PAX2 IM markers in MP cells by comparing their fluorescence intensity to hiPSC markers through immunofluorescence analysis (Supplementary Figure 1D).

**FIGURE 4:**
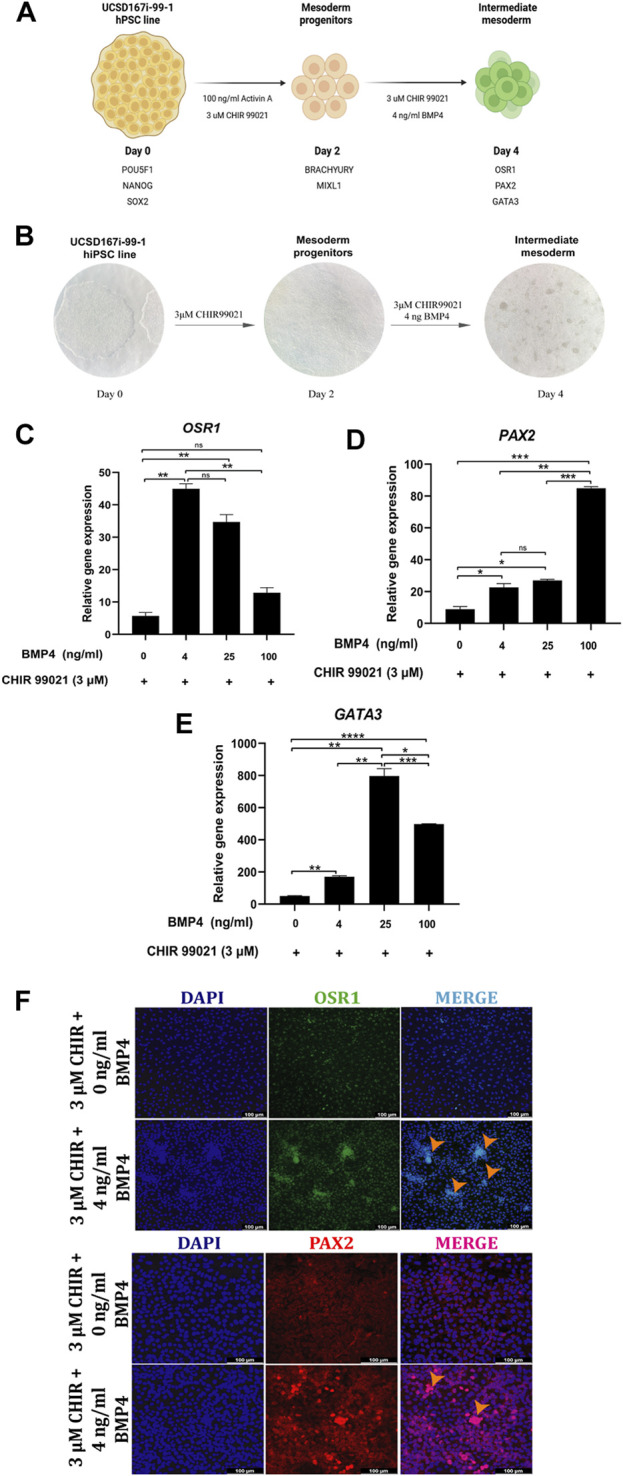
BMP and WNT-mediated IM cells differentiation. **(A)** Schematic diagram of the modified differentiation strategy, without Activin A and low BMP4 concentrations, to induce hiPSC into MP and IM cells. **(B)** Representative bright-field images showing the stages of cell differentiation from hiPSC to IM cells. **(C–E)** Relative mRNA expression levels of IM markers *OSR1*, *PAX2* and *GATA3* determined by RT*-*qPCR in differentiated cells after 48 h of treatment with 0, 4, 25 and 100 ng/mL BMP4 and 3 µM CHIR99021. **(F)** Immunofluorescence images of cells stained for IM markers OSR1, PAX2, merged with nucleus marker DAPI (cyan or magenta) at 48 h of treatment with 4 ng/mL BMP4 and 3 µM CHIR99021. Scale bars: 100 μm. Orange arrowheads indicate cells with a spheroid morphology, which is characteristic of IM cells. Error bars represent mean *±* SEM, *n* = 3 independent experiments, n. s not significant, *p < *0.05*, **p < 0.01, ***p < 0.001, ****p < 0.0001. Gene expression levels were determined by relative quantification compared to undifferentiated hiPSC. hiPSC human induced pluripotent stem cells, MP mesoderm progenitor, IM intermediate mesoderm, DE definitive endoderm.

RT-qPCR of CHIR- and BMP4-induced cells revealed that BMP4 treatment significantly upregulated the expression of IM genes (*OSR1*, *PAX2*, and *GATA3*) compared to CHIR-induced cells (Figures 4C–E). However, BMP4 treatment decreased *OSR1* and increased *PAX2* gene expression in a dose-dependent manner (Figures 4C, D).

Hence, we chose an optimal BMP4 treatment of 4 ng/mL and 3 μM CHIR99021 for 48 h in further experiments.

Immunofluorescence analysis detected PAX2 and OSR1 in BMP4 treatment at 4 ng/mL and 3 μM CHIR99021, and spheroid-forming cells were observed (Figure 4F).

**FIGURE 5:**
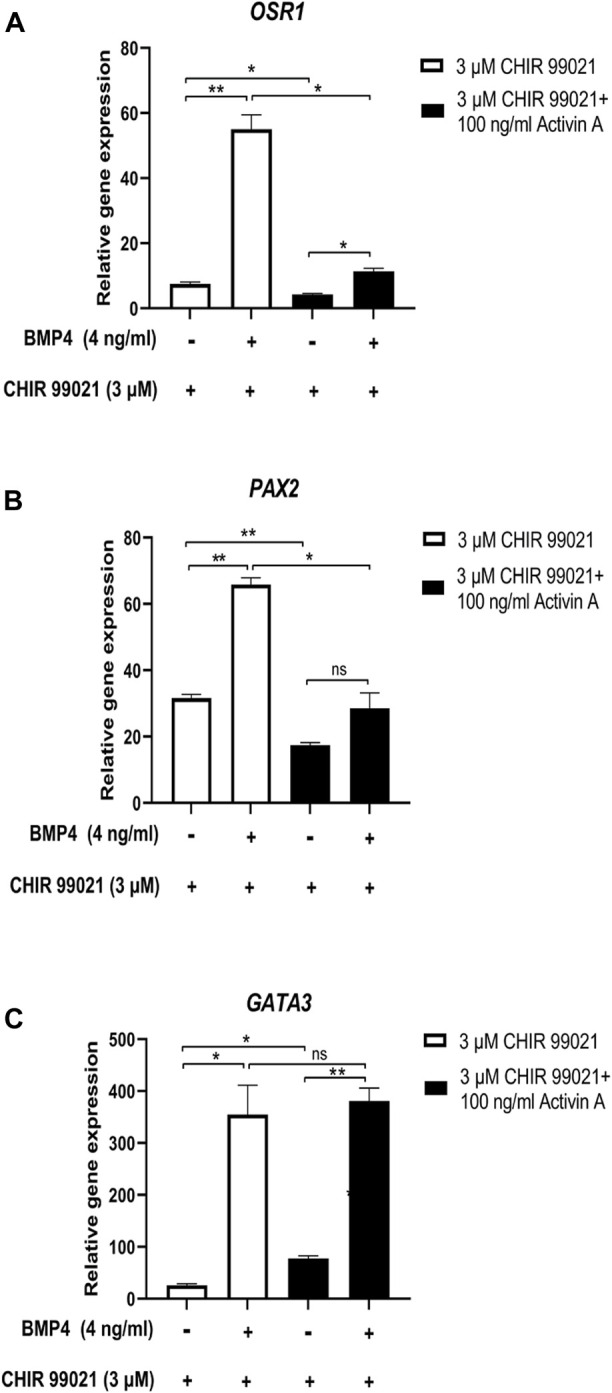
Effect of Low-Dose BMP4 on Nodal/WNT-Induced IM cells differentiation. **(A–C)** Relative mRNA expression levels of IM markers *OSR1*, *PAX2* and *GATA3* determined by RT*-*qPCR in differentiated cells at 48 h of treatment with 0 and 4 ng/mL BMP4 and 3 µM CHIR99021, and differentiated from MP cells treated with 3 µM CHIR99021 alone and with 100 ng/mL Activin A. Error bars represent mean *±* SEM, *n* = 3 independent experiments, n.s. not significant, *p < *0.05*, **p < 0.01, ***p < 0.001, ****p < 0.0001. Gene expression levels were determined by relative quantification compared to undifferentiated hiPSC. hiPSC human induced pluripotent stem cells, MP mesoderm progenitor, IM intermediate mesoderm, DE definitive endoderm.

Additionally, we investigated the effect of low doses of BMP4 (4 ng/mL) on MP cells differentiated with 100 ng/mL Activin A, alone or with 3 μM CHIR99021 and studied *OSR1*, *PAX2* and *GATA3* IM markers by RT-qPCR (Figures 5A–C). MP cells treated with 100 ng/mL Activin A and 3 μM CHIR99021 showed a significantly decreased expression of the IM markers *OSR1* and *PAX2* compared to cells treated with CHIR99021 alone (Figures 5A, B). However, we did not observe differences between treatments in the expression of the IM marker *GATA3* (Figure 5C).

## Discussion

Here, we report an efficient and reproducible protocol for generating IM cells from the UCSD167i-99-1 hiPSC line (Panopoulos et al., 2017). Our protocol faithfully recapitulates key molecular events of IM development described in the current embryological literature. We achieved this by optimizing the signaling factors Nodal, WNT, and BMP. Unlike existing methods that often rely on combined high Nodal and WNT activation typically associated with the anterior primitive streak (APS) origin, our protocol emphasizes WNT activation from a PPS origin, mimicking the natural developmental process of IM cells. Furthermore, we employ low BMP4 concentrations, compared to protocols using high BMP4 that can lead to undesired cell fate transitions. This aligns with the established role of BMP4 gradients in separating IM and LPM domains during development. Current IM differentiation protocols exhibit significant heterogeneity in culture conditions and utilized markers. Our protocol addresses this by focusing on optimizing the signaling pathways crucial for IM development, while acknowledging the limitations of current marker identification. Importantly, our method reduces the number and concentrations of morphogens compared to other protocols, while maintaining high yields of IM cells. Many established protocols require various morphogens or concentrations (Yucer et al., 2017; Miyazaki et al., 2018; Knarston et al., 2020; Bejoy et al., 2022; Gong et al., 2022), leading to inconsistent target cell generation and lacking a lineage-directed approach. By replicating features of embryogenesis not captured in previous protocols, our method offers a more robust, reproducible, and efficient approach for organoid development and disease modeling.

An essential parameter in optimizing the differentiation of MP cells from the UCSD167i-99-1 hiPSC line was the concentration of CHIR99021. Our results showed that high concentrations of CHIR99021 (>4 μM) achieve robust commitment of the UCSD167i-99-1 hiPSC line to TBXT+/MIXL1+ MP cells within 48 h, with a reduction in pluripotency. However, we observed a decrease in the number of cells at > 4 μM doses. This decrease aligns with previous findings on the detrimental effects of DMSO, a commonly used solvent in drug screening, on cell morphology and attachment, and viability in a dose-dependent manner (Pal et al., 2012). In addition, our results demonstrated a significant upregulation of both *TBXT* and *MIXL1* expression in hiPSC treated with 3 μM and 4 μM CHIR99021 for 48 h, highlighting the potency of 3 μM CHIR99021 in inducing MP differentiation markers, as it achieved comparable levels of induction to a higher concentration >4 μM without compromising cell abundance. Conversely, the pluripotency markers *POU5F1* and *NANOG* showed a significant downregulation in hiPSC treated with 3 μM and 4 μM CHIR99021 compared to the 2 μM treatment group. Therefore, we opted for 3 μM CHIR99021 as the optimal concentration, balancing efficient differentiation with maintaining a robust cell population. Notably, this selection aligns with established protocols for MP differentiation using CHIR99021 in UCSD167i-99-1 hiPSC, typically employing a 3 μM concentration (Musah et al., 2017; Bejoy et al., 2021). This further validates the use of CHIR99021 as a potent activator of WNT signaling in UCSD167i-99-1 hiPSC.

Furthermore, the persistence of levels of pluripotency markers during mesendoderm specification aligns with previous reports (Niwa et al., 2000; Teo et al., 2011; Radzisheuskaya et al., 2013).

Another important finding of our research was that removing the Nodal pathway agonist yielded the most reproducible and efficient MP cell differentiation. In this regard, we observed that the presence of Nodal signaling, along with WNT signaling, increased the expression of the DE marker *SOX17* and *FOXA1*. This finding is consistent with the observation that the Nodal signaling pathway has been described to differentiate hiPSC into DE cells in several induction protocols (Ardila Riveros et al., 2021; D’Amour et al., 2005; McLean et al., 2007; Pagliuca et al., 2014).

However, while previous reports (Fleming et al., 2013) highlight the role of low Nodal signaling in modulating BMP signaling, which is essential for IM formation, current attempts at IM induction from hiPSC rely on high levels of Nodal signaling and WNT activation to specify mesoderm with an origin in the PPS, which is not consistent with the embryological literature (Yucer et al., 2017; Bejoy et al., 2022; 2021). According to scientific literature, during gastrulation, a subset of epiblast cells invaginate through the primitive streak to form the endoderm and mesoderm (Tam and Behringer, 1997; Loebel et al., 2003). The APS generates DE (Levak-Švajger and Švajger, 1974; Takasato et al., 2014; Ardila Riveros et al., 2021), whereas the PPS forms mesoderm (Tam and Beddington, 1987; Lawson et al., 1991; Takasato et al., 2014; Takasato and Little, 2015; Ardila Riveros et al., 2021), and mesoderm will give rise to the axial, paraxial, intermediate, and lateral plate mesoderm (Prummel et al., 2020).

On the other hand, the fact that WNT pathway activation alone efficiently drives hPSC into mesodermal lineages but also specifies endoderm lineage is in line with the shared primitive streak origin of both germ layers during gastrulation, suggesting that these cells may represent mesendoderm progenitors (Levak-Švajger and Švajger, 1974; Tam and Beddington, 1987; Lawson et al., 1991). Indeed, DE and MP co-express *TBXT*, *MIXL1*, *SOX17*, *FOXA2*, among other markers (Kubo et al., 2004; Tada et al., 2005; Faial et al., 2015).

Regarding IM differentiation, our data demonstrated that BMP4 ligand could efficiently achieve IM differentiation at all concentrations tested using the UCSD167i-99-1 hiPSC line, aligning with the established role of BMP signaling in regulating mesodermal cell fate and specifically differentiating them into IM (Knarston et al., 2020; Magro-Lopez and Muñoz-Fernández, 2021). Notably, low doses of BMP4 efficiently promoted the differentiation of MP cells into OSR1+ IM cells, while high doses repressed IM differentiation. In line with this, prior studies have shown that mesodermal subtypes are specified along the mediolateral axis depending on BMPs activity, with low activity enhancing IM formation and higher activity in the LPM tissue (Tonegawa and Takahashi, 1998; James and Schultheiss, 2005; Knarston et al., 2020). However, several existing protocols for IM induction still employ high doses of BMP4 (Yucer et al., 2021; 2017).

In contrast, we did not observe a similar pattern for PAX2 and GATA-3 marker expression. Interestingly, previous studies have identified OSR1 as an early marker of IM (James et al., 2006), while PAX2 and GATA3 have been widely used as late markers in the nephric duct, an epithelial duct differentiated from IM cells and essential for all further urogenital development (Grote et al., 2006; Boualia et al., 2013; Morizane and Lam, 2015; Sanchez-Ferras et al., 2021). Furthermore, we found that low-dose BMP4 treatment enhanced IM differentiation when MP cells were differentiated by WNT activation alone. This result suggests that IM differentiation may be optimized if it originates from PPS (WNT activation alone) rather than APS (combined Nodal and WNT activation) (Takasato et al., 2016; Knarston et al., 2020), supporting our previous results.

These findings highlight the heterogeneity not only in the culture protocols employed in current differentiation methods but also in the markers utilizied for identifying the mesoderm lineage. However, future efforts should focus on identifying more specific markers for early IM stages to enhance differentiation accuracy. In summary, optimizing the Nodal, WNT and BMP signaling pathways in a UCSD167i-99-1 hiPSC line, significantly enhanced the induction of IM cells from hPSC compared to existing protocols. We found the need to precisely define the specification of IM from hPSC and establish a standardized differentiation protocol that closely recapitulates *in vivo* IM development to efficiently generate human organoids that closely resemble the human embryo in both identity and function. Further advancements in IM protocols may involve exploring the impact of different Activin/Nodal signaling levels, alongside integrating other morphogenic signaling pathways and investigation of short-term culturing strategies. These approaches warrant further investigation to expand our understanding of human development and disease, enabling the development of innovative therapies for a wide range of conditions.

## Data Availability

The raw data supporting the conclusion of this article will be made available by the authors, without undue reservation.

## Supplementary material

Supplementary Figure 1 Negative controls of IM markers in MP cells. **(A, B, C)** Relative mRNA expression levels of IM markers OSR1, GATA3 and PAX2 determined by RT-qPCR analysis in MP cells at 48 h of treatment with 100 ng/ml Activin A and 3 μM CHIR99021. The reference line at level 1 of expression indicates the baseline expression level in undifferentiated hiPSC (control). **(D)** Immunofluorescence images of undifferentiated hiPSC colonies and MP cells stained for IM markers OSR1 (green), PAX2 (red), merged with nucleus marker DAPI (cyan or magenta) at 48 h of treatment with 3 μM CHIR99021. Scale bars: 100 μm. Error bars represent mean ± SEM, n=3 independent experiments, n.s. not significant, *P < 0.05, **P < 0.01, ***P < 0.001, ****P < 0.0001. Gene expression levels were determined by relative quantification compared to undifferentiated hiPSC. hiPSC human induced pluripotent stem cells, MP mesoderm progenitor, IM intermediate mesoderm, DE definitive endoderm.
