# Correction: Optimizing Nodal, Wnt and BMP signaling pathways for robust and efficient differentiation of human induced pluripotent stem cells to intermediate mesoderm cells

**DOI:** 10.3389/fcell.2025.1655115

**Published:** 2025-07-11

**Authors:** Esmeralda Magro-Lopez, Elena Vazquez-Alejo, María de la Sierra Espinar-Buitrago, María Ángeles Muñoz-Fernández

**Affiliations:** ^1^ Molecular Immuno-Biology Laboratory, Immunology Section, Hospital General Universitario Gregorio Marañón (HGUGM), Madrid, Spain; ^2^ Instituto de Investigación Sanitaria Gregorio Marañón (IiSGM), Madrid, Spain; ^3^ Biomedical Research Networking Center in Bioengineering, Biomaterials and Nanomedicine (CIBER-BBN), Instituto de Salud Carlos III (ISCIII), Madrid, Spain

**Keywords:** hiPSC, mesoderm progenitors, intermediate mesoderm, Nodal, Wnt, BMPs, 3D organoids, urogenital organoids

In the published article, there was an omission in the Funding statement.

The co-funders Ministerio de Ciencia e Innovación and Unión Europea–European Regional Development Fund were erroneously omitted.

