# Supplementary figures and images for: Optimizing Nodal, Wnt and BMP signaling pathways for robust and efficient differentiation of human induced pluripotent stem cells to intermediate mesoderm cells
Source: Front Cell Dev Biol. 2024 Jun 3;12:1395723. doi: 10.3389/fcell.2024.1395723 (PMC11182123; doi:10.3389/fcell.2024.1395723)

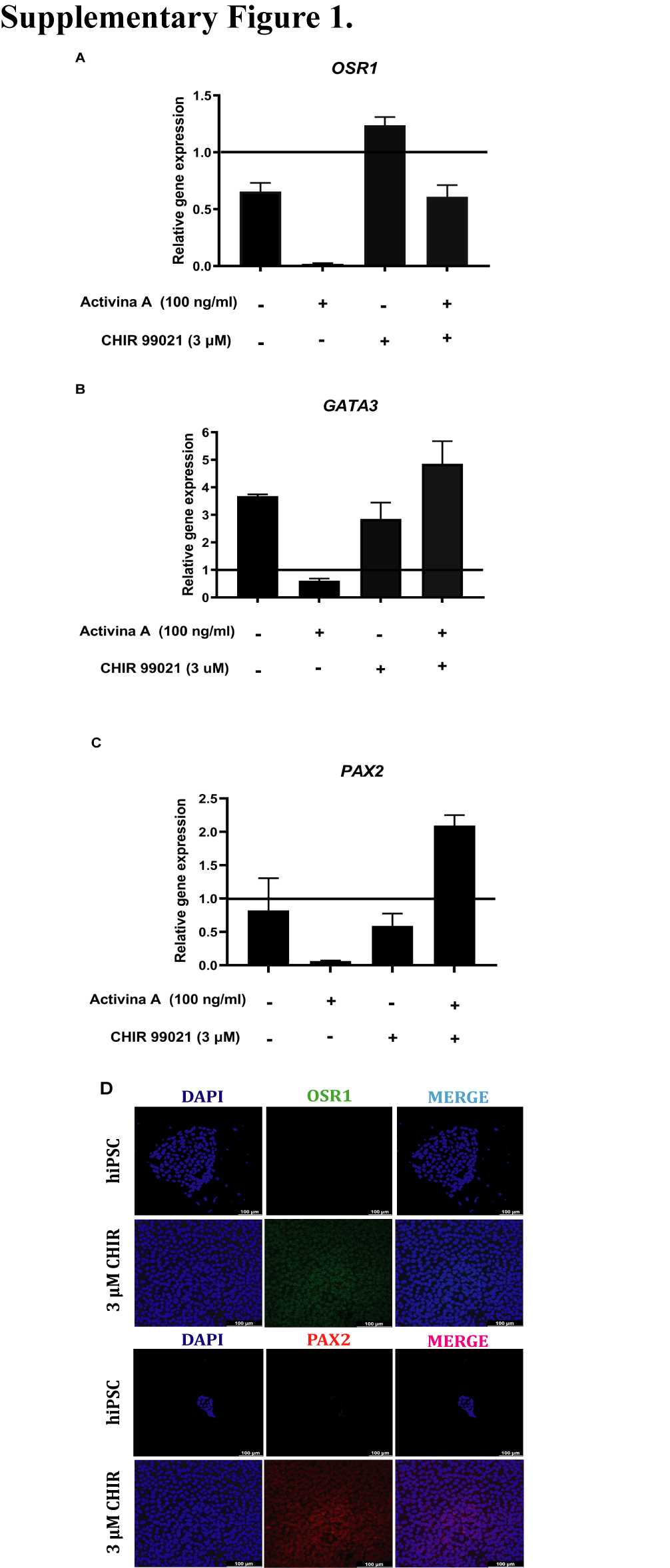

Supplement: Supplementary file 1 [file Image1.tif]
